# The effect of injection speed and serial injection on propidium iodide entry into cultured HeLa and primary neonatal fibroblast cells using lance array nanoinjection

**DOI:** 10.1186/s40064-016-2757-5

**Published:** 2016-07-15

**Authors:** John W. Sessions, Tyler E. Lewis, Craig S. Skousen, Sandra Hope, Brian D. Jensen

**Affiliations:** Department of Mechanical Engineering, Brigham Young University, Provo, UT 84602 USA; Department of Microbiology and Molecular Biology, Brigham Young University, Provo, UT 84602 USA

**Keywords:** Lance array nanoinjection, Speed of injection, Serial injection, Injection-dose response

## Abstract

**Background:**

Although site-directed genetic engineering has greatly improved in recent years, particularly with the implementation of CRISPR-Cas9, the ability to deliver these molecular constructs to a wide variety of cell types without adverse reaction is still a challenge. One non-viral transfection method designed to address this challenge is a MEMS based biotechnology described previously as lance array nanoinjection (LAN). LAN delivery of molecular loads is based upon the combinational use of electrical manipulation of loads of interest and physical penetration of target cell membranes. This work explores an original procedural element to nanoinjection by investigating the effects of the speed of injection and also the ability to serially inject the same sample.

**Results:**

Initial LAN experimentation demonstrated that injecting at speeds of 0.08 mm/s resulted in 99.3 % of cultured HeLa 229 cells remaining adherent to the glass slide substrate used to stage the injection process. These results were then utilized to examine whether or not target cells could be injected multiple times (1, 2, and 3 times) since the injection process was not pulling the cells off of the glass slide. Using two different current control settings (1.5 and 3.0 mA) and two different cell types (HeLa 229 cells and primary neonatal fibroblasts [BJ(ATCC^®^ CRL-2522™)], treatment samples were injected with propidium iodide (PI), a cell membrane impermeable nucleic acid dye, to assess the degree of molecular load delivery. Results from the serial injection work indicate that HeLa cells treated with 3.0 mA and injected twice (×2) had the greatest mean PI uptake of 60.47 % and that neonatal fibroblasts treated with the same protocol reached mean PI uptake rates of 20.97 %.

**Conclusions:**

Both experimental findings are particularly useful because it shows that greater molecular modification rates can be achieved by multiple, serial injections via a slower injection process.

## Background

Gene therapy and gene medicine approaches to correcting disease represent a major paradigm shift in how clinicians are able to help patients, moving from a framework of reactionary treatment of disease manifestations to fundamental, proactive prevention of genetic alterations causing the disease (Byrne et al. 2012; Griesenbach et al. 2015; Shimamura et al. 2013). While still in a relatively early stage of development, medical approaches designed to engineer genetic outcomes have had promising results in terms of both monogenic (Bainbridge et al. 2008; Hauswirth et al. 2008; Jacobson et al. 2012; Maguire et al. 2008; Patel et al. 2014; Sahel and Roska 2013; Yla-Herttuala 2012) and polygenic (Jessup et al. 2011; Kochenderfer and Rosenberg 2013; Kranias and Hajjar 2012; Sikkel et al. 2014; Simonato et al. 2013; Zsebo et al. 2014) disease corrections.

Unfortunately, the actual method for transmission of the genetic loads to target cells remains a challenge (Doherty and McMahon 2009; Khalil et al. 2006; Lukacs et al. 2000; Mellott et al. 2013). Many biotechnologies have been created to help address this issue (with mixed results) (Choi et al. 2010; Jen et al. 2004; Lin and Huang 2001; Lin et al. 2001; Wiethoff and Middaugh 2003). The primary goal of all of these methods is to site-direct genetic loads into cells without harming the host systemically or the target cell locally (Mellott et al. 2013). Key features frequently noted as critical design requirements for these biotechnologies include:

- High transfection efficiency
- Effective in a wide range of cell types
- Flexible pay load capacity
- No immunologic response
- No insertional mutagenesis

**Fig. 1:**
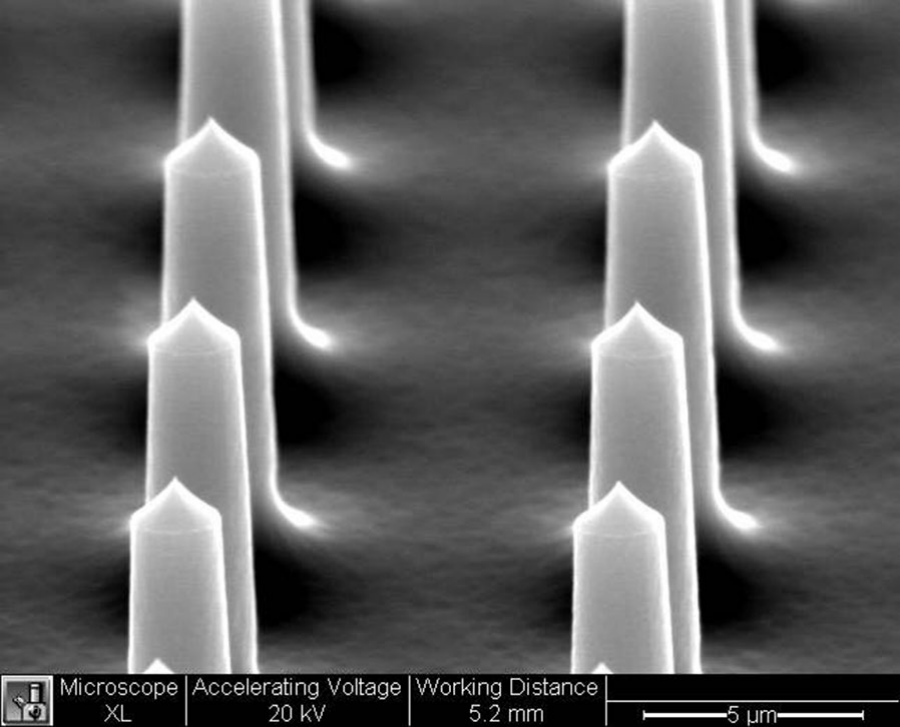
SEM image of *two rows* of lances contained on the lance array silicon chip. Lances measure 10 μm in length, 1–2.5 μm in diameter, and spaced 10 μm from center to center

One non-viral transfection biotechnology, known as lance array nanoinjection (LAN), has been created with these design requirements in mind. LAN works by using a combination of physical penetration of target cell membranes and electrical delivery of molecular loads using a microfabricated silicon etched array of lances (Lindstrom et al. 2014; Teichert et al. 2013). Figure 1 shows an SEM image of a lance array which contains 10 μm length lances spaced 10 μm from center to center in a grid pattern, ultimately forming 4 million lances on a 2 by 2 cm chip.

**Fig. 2:**
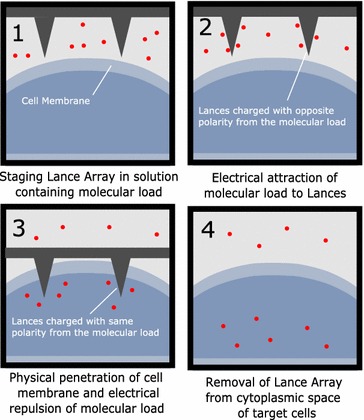
Lance array nanoinjection stepwise process. **1** Staging the lance array in the solution containing the desired molecular load. **2** Electrical attraction of the molecular load onto the lances. **3** Physically penetrating the cell membrane of target cells and electrical repulsion of the molecular load into the cytoplasmic space. **4** Removal of the lance array, leaving the molecular load in the intracellular space of target cells

Procedurally, nanoinjection works in a series of four major steps which include: staging the lance in the solution containing the desired molecular load, electrical attraction of the molecular load onto the lance, physical penetration of the cell membrane of target cells and electrical repulsion of the molecular load into the cytoplasmic space, and finally removal of the lance (Aten et al. 2011, 2012; Wilson et al. 2013) (see Fig. 2 for illustration of LAN process).

There are several attractive features of LAN relative to other transfection methods. First, it does not rely on delivery agents that can cross-react with the immune system [such is the case with several viruses (Follenzi et al. 2007; Matrai et al. 2010; Mellott et al. 2013; Ritter et al. 2002; VandenDriessche et al. 2002)], nor does it create cytotoxic effects in target cells [such is the case with many chemical based methods (Mellott et al. 2013; Wiethoff and Middaugh 2003)]. Second, because the lances are 1–2.5 μm in diameter, the resulting pores created during the injection event are relatively large, making it possible for large molecules to transiently pass into the cell. Even though the pores are relatively large, the trauma induced during the process is relatively minimal, as evidenced by high cell viability rates (78–91 %) previously noted (Lindstrom et al. 2014). This latter feature of cell viability is an issue in some instrumentation based transfection methods, such as electroporation (Barsoum 1995) and microinjection (Aten et al. 2012).

Despite these attractive features of LAN, one short-coming that LAN as well as all non-viral transfection technologies encounter is that transfection rates are lower than what can be achieved with viral modalities (Mellott et al. 2013). This work seeks to directly address this challenge related to efficient molecular delivery by considering two intertwined procedural variables unique to LAN which include: the speed of injection and serial injection of the same sample. In prior testing, it has been noted that following a single injection event, many cells do not stay adherent to the glass slide used for staging the injection process. The purpose of investigating the effect of speed of injection is to determine the extent that cell removal can be minimized such that serial injection protocols can be investigated.

Indeed, it is shown in this work that by slowing the speed of the injection process that target cells are able to remain adherent to the glass slide using for staging the injection. Because the cells remain post-injection, it is possible to inject multiple times and thereby increase the amount of molecular load delivered to the cell.

To help establish the robustness of this procedural investigation, as part of the serial injection testing, two different cell types, immortalized HeLa culture and primary neonatal fibroblast cells, were used to determine how the different cell types respond. To demonstrate molecular load delivery, propidium iodide (PI), a dye typically impermeable to the cell membrane, was used in conjunction with flow cytometry to quantify the injection-dose response.

Because the speed of injection experimentation led to serial injection experimentation, the following will be compartmentalized to consider the speed of injection work first, followed immediately by the serial injection work.

## Methods: General

This work consists of two major experiments—the speed of injection and serial injection experiments. The speed of injection work, which is presented first, is a precursor experiment that led to further exploration that makes up the serial injection experiment. Both used common experimental elements which are detailed in this general methods section. For experimental elements unique to the specific experiment, separate descriptions are provided.

### Lance array fabrication

Reference (Teichert et al. 2013) provides a complete description of the microfabrication process used for creating the silicon lance array and is presented here simply for convenience. The lance array microfabrication process consisted of using positive photoresist (AZ330F) to pattern a grid of circles that became pillar-like structures following deep reactive ion etching (DRIE). These pillars were then treated with a sulfur hexafluoride (SF6) isotropic plasma etch, which serves to form a pointed tip on the pillars, resulting in lances (see Fig. 1).

### Injection set-up

**Fig. 3:**
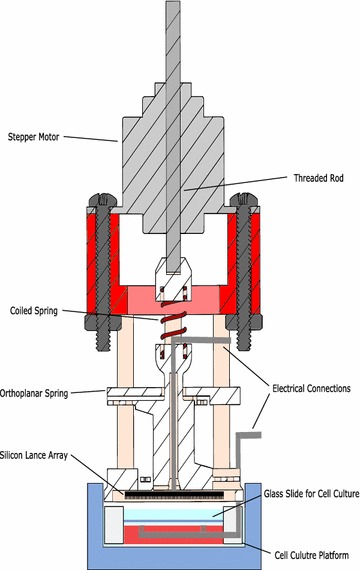
Cross-sectional schematic of injection device. Components include (*top*–*bottom*): stepper motor, threaded rod, coiled and orthoplanar springs, electrical connections, silicon lance array, glass slide for cell culture, and cell culture platform

**Fig. 4:**
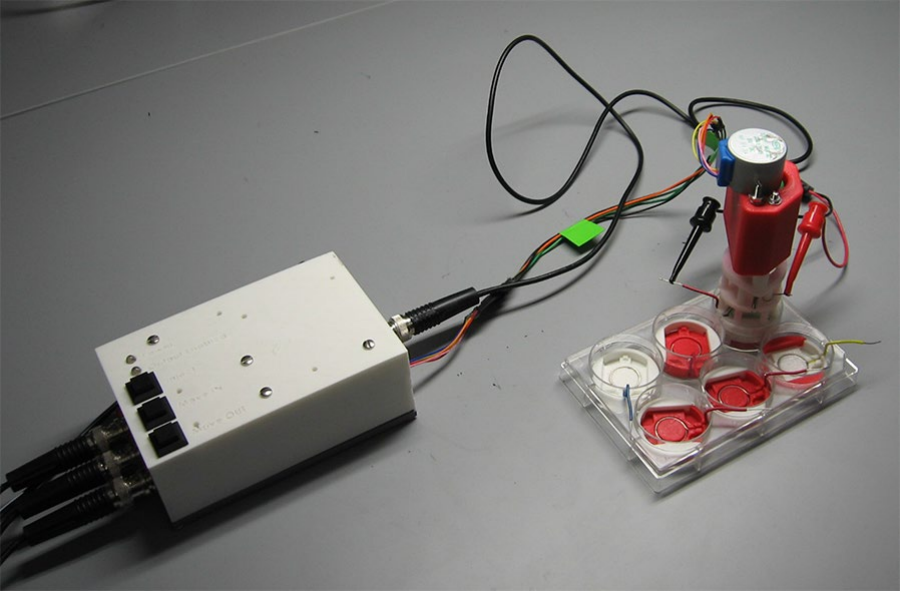
Experimental set-up showing the electrical control box receiving three separate input signals coming from three power supplies (not shown) and outputting appropriately timed output signals to the injection device mounted above the prepared six-well plate. Cell culture platforms with the prepared cell cultures are seen as *white* and *red circular* components resting in the wells of the six-well plate

Figure 3 shows a schematic, cross-sectional view of the injection device and contains eight major components which include: stepper motor, threaded rod, coiled spring, orthoplanar spring, electrical connections, silicon lance array, glass slide for the cell culture, and cell culture platform. The stepper motor and electrical connections receive input signals from the electrical control box (see Fig. 4). During the injection process, the stepper motor causes the threaded rod to vertically displace the coiled and orthoplanar springs. The lance array is attached to the inferior surface of the orthoplanar spring and interacts with the cell culture contained on the glass slide according the process outlined in Fig. 2. The cell culture platform serves to facilitate alignment with the orthoplanar spring and also helps fix the glass slide, preventing it from adhering to the silicon lance array.

### Electrical control box

**Fig. 5:**
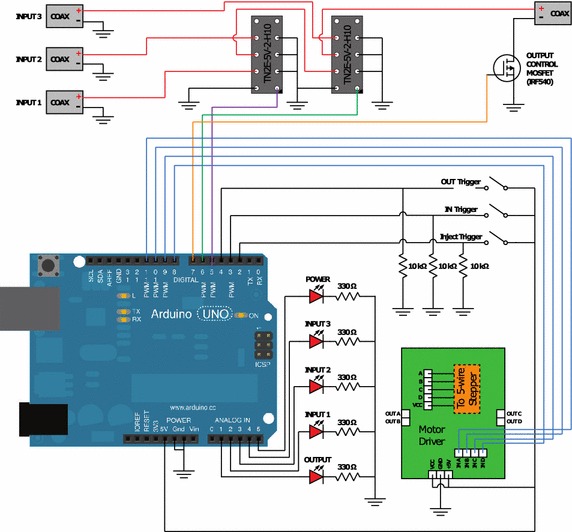
Electrical schematic for the current control box. An Arduino was used to control two relays and a stepper motor driver for the injection process. Five LEDs are used as indicators for power, output, and which input being passed through the box

The electrical control box, which provides electrical input to the injection device, operates by receiving three inputs from three separate power supplies (2400 SourceMeter, Keithley) (see Fig. 4). Figure 5 is a full electrical schematic of the electrical control box where it illustrates electrical inputs from the power supplies running through two relays arranged in series. The first relay was used to allow either Input 1 or 2 to pass, while the second relay was used to allow the input from the previous relay or Input 3 to pass. This allowed one input at a time to pass using two digital pins (one to control each relay, using 5 V or GND).

The output signals from the electrical control box used for the serial injection testing can be described as the following: Output 1, current controlled of either 1.5 or 3.0 mA for 20 s; Output 2, 10 intermittent pulsing events between 0 and +7 V for 20 ms (2 ms period); Output 3, 5 s interval of +1.5 V. In addition to these actions, the electrical control box also operates the stepper motor attached to the superior end of the injection device. It should be noted, Outputs 2 and 3 occur when the lances are inserted into the cells.

## Methods: Speed of injection experimentation

### Stepper motors

Two separate stepper motors [28BYJ-48-5V, Rohs; TSFNA25-150-17-023-LW4, Anaheim Automation (AA)] were used to for the speed of injection testing in order to achieve the speeds needed and still ensure that the stepper motors would not skip steps by being driven too quickly. Five different speeds were used for this experiment, which include 0.08, 0.16, 0.60, 1.80, 3.00 mm/s. For the slower speeds of 0.08 and 0.16 mm/s, the Rohs stepper motor was used and for 0.60, 1.80, and 3.00 mm/s speeds, the AA stepper motor was used. The injection device was the same for all sets of injection tests, with the exception of the stepper motor being changed to match speed conditions.

### Cell culture preparation

For all experiments, untreated glass coverslip slides were used as the substrate for seeding cells. HeLa 229 cells were plated on glass slides within six-well plates (Sarstedt) and incubated at 37 °C and 5 % carbon dioxide. Culture media contained Dulbeccos Modified Eagles Medium (DMEM, Gibco) with 10 % Fetal Bovine Serum (FBS, Denville Scientific) and streptomycin/penicillin (Gibco). Cell cultures were allowed to incubate for 24 h following the plating process to ensure adequate adherence to the glass slide. Following the 24 h, a mono-layer of HeLa cells, which were approximately 70 % confluent, had their growth media removed and were re-supplied with 2 mL of phosphate buffered solution (PBS, Gibco) in final preparation for injection.

### Treatment protocols

The following describes the various sample types used in the speed of injection testing:

- *Non-Treatment Control (NTC)*: Received no lancing.
- *Treatment Protocol for Different Speeds*: Samples were lanced a single time and received no applied voltage during the injection process. Five different injection speeds were applied during the injection event and include: 0.08, 0.16, 0.60, 1.80, 3.00 mm/s.

As a convention, specific treatment sample types will be designated by the speed of injection. For example, HeLa/0.08 refers to a treatment sample that was lanced a single time at 0.08 mm/s.

### Post-treatment analysis

After the injections were completed, all samples were given 0.5 mL of 5× trypsin (Gibco) and incubated 5 min at 37 °C to facilitate removal of the cells from the glass slides. After the 5 min, samples were treated with 1.5 mL of DMEM/FBS media to deactivate the trypsin and then centrifuged at 2000 rpm for 10 min. The supernatant was then removed, 0.25 mL of PBS was added to each sample, and each sample was then vortexed to prepare them for hemocytometry. Quantification of the number of cells were performed according to standard hemocytometry methods using trypan blue (Caprette 2012).

### Statistical analysis

After cell counts were obtained using hemocytometry, data for treatment samples were normalized relative to the Non-Treatment Controls according to the following formula.

1 $$\frac{Cell\,Count\,in\,Treatment\, Sample}{{Cell\,Count\,in\,Non{\hbox{-}}Treated \,Control\,\left( {NTC} \right)}}$$

This normalized data was then statistically analyzed in JMP (SAS), using initially an ANOVA test to screen for statistically significant relationships (F-ratio: 16.5426) followed by individual student t-tests (α = 0.05).

## Results and discussion: Speed of injection experimentation

**Fig. 6:**
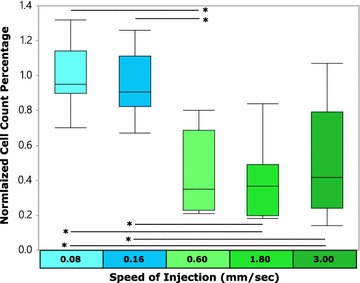
Injection speed *box plot*. The *two left-most box plots* were the result of the slower stepper motor (Rohs) whereas the *three right-most box plots* were the result of the faster stepper motor (AA). Statistically significant relationships are noted with an asterisk

Figure 6 shows a set of box plot results for the data generated for the five different injection speeds. The left-most two box plots were obtained by using the slower stepper motor (Rohs), while the other three box plots were obtained from the slightly faster stepper motor (AA). It can be seen that statistically significant relationships exist in all cases between the slower and faster stepper motors (<0.0001) (see Table 1).

**Table 1:** P values for speed of injection experiment

Comparison	P value	Comparison	P value
HeLa/0.08 versus HeLa/0.16	0.6466	HeLa/0.16 versus HeLa/1.80	<0.0001
HeLa/0.08 versus HeLa/0.60	<0.0001	HeLa/0.16 versus HeLa/3.00	<0.0001
HeLa/0.08 versus HeLa/1.80	<0.0001	HeLa/0.60 versus HeLa/1.80	0.7180
HeLa/0.08 versus HeLa/3.00	<0.0001	HeLa/0.60 versus HeLa/3.00	0.4362
HeLa/0.16 versus HeLa/0.60	<0.0001	HeLa/1.80 versus HeLa/3.00	0.2566

Table 2 provides a summary of the results showing that for both the 0.08 and 0.16 mm/s speeds, that the mean normalized cell count is more than double the highest normalized mean obtained with the faster stepper motor.

**Table 2:** Statistical summary for injection speed experiment

Injection speed (mm/s)	Sample size (n)	Mean normalized cell count
HeLa/0.08	10	0.993
HeLa/0.16	10	0.946
HeLa/0.60	10	0.427
HeLa/1.80	10	0.390
HeLa/3.00	10	0.507

Suggested in these results is that the cultured cells are able to adhere better to the glass surface used for staging the injection when using injection speeds of 0.08–0.16 mm/s. These findings are consistent with atomic force microscopy (AFM)-based single-cell force spectroscopy (SCFS) studies (Benoit et al. 2000; Evans and Calderwood 2007; Florin et al. 1994; Helenius et al. 2008; Hong et al. 2012; Lehenkari and Horton 1999; Zhang et al. 2002). Friedrichs et al. (2013) demonstrated with a single cell adhered to an AFM probe by cell adhesion molecules (CAMs), that when placed in contact with a substrate surface, the speed with which the AFM probe was removed was directly related to the viscous forces created between the substrate surface and the cell. If sufficiently strong viscous forces were generated by removing the AFM probe (with the attached cell) fast enough, the cell would experience a series of five major events which include:

1. Shrinking of the surface area contact between the cell and substrate surface.
2. Cell body removal from the substrate with only membrane nanotubes linking the cell and substrate.
3. Stressing of the CAM linkages between the cell and AFM probe—reaching a non-linear maximum level as cytoskeletal structures are strained in the direction of motion.
4. Peripheral rupture of the CAM structures leading to a rapid decrease in adhesion force between the cell and the AFM probe.
5. Cell removal from the AFM probe as the viscous effects from the substrate-cell interface overcome the cellular adhesion to the AFM probe.

Returning to the context of LAN, it is suggested that for the majority of cells present on the glass slides that experience injection speeds of ≤0.16 mm/s, that the viscous forces created by the removal of the silicon lance array does not exceed the strength with which the cells are adhered to the glass slide. For treatment samples experiencing greater speeds of lance array removal, it appears that fewer cells are able to withstand these removal forces and are pulled away from the glass.

## Methods: Serial injection experimentation

Using the fact that slowing the injection process results in an increase in cell number remaining on the glass slide substrate used for staging the injection process, it was proposed to investigate whether or not target cells could be injected multiple times and exhibit an increase in molecular load delivery. Furthermore, it was also proposed to determine whether a difference exists in molecular delivery when using different cell types. To explore this latter item, two cell types were selected which include HeLa 229 cells (commonly used in basic research) and primary, human neonatal fibroblasts [BJ(ATCC^®^ CRL-2522™)] (used in wound healing applications).

For convenience, the injection speed of 0.16 mm/s was used for the serial injection testing because it was twice as fast as the 0.08 mm/s speed setting and still had a high mean number of cells still adherent to the glass slide following injection (i.e. 94.6 %).

### Cell culture preparation

For both cell types, test preparation began with seeding glass slides contained within six-well plates with 2 mL of a growth media, which consisted of: Dulbeccos Modified Eagles Medium (DMEM, Gibco), 10 % Fetal Bovine Serum (FBS, Denville Scientific), and streptomycin/penicillin (Gibco). Cells were incubated overnight prior to injection at standard conditions of 37 °C and 5 % carbon dioxide.

Following this process, glass slides were transferred to new six-well plates and snapped into 3D printed cell culture platforms (used to help align the injection device). Cells, now staged for the injection process, were given 2 mL of phosphate buffered solution (PBS, Gibco) per well. At this point, both cell types had formed a mono-layer of approximately 70 % confluency.

### Propidium iodide

Propidium iodide (Sigma-Aldrich) was used as a molecular marker in this experiment because it is typically impermeable to the cell membrane. Because the lances used in LAN penetrate target cells and then deliver PI to the intracellular space of these target cells, it is an indicator of successful delivery. Once in the cell, PI can intercalate will nucleic acids, which results in fluorescent activity 20–30 times greater than normal, thereby providing a detectable means of transfection rates during flow cytometry.

### Treatment protocols

Treatment samples were generated using the following protocols for both HeLa and Fibroblast cells which include:

- *Non-Treatment Control* (*NTC*): Received no lancing, no applied voltage, and no PI.
- *Background Control for PI* (*BC*): Received no lancing, no applied voltage. Received 0.02 mg/mL PI.
- *Treatment Protocol 1.5 or 3.0* *mA, injected 1, 2, or 3 times*: Lanced, receive 0.02 mg/mL PI, and received current control which consisted of: 20 s application of either 1.5 or 3.0 mA, followed by 10 intermittent pulsing events between 0 and +7 V for 20 ms (2 ms period), followed by 5 s of a +1.5 DC voltage.

The parameters for treatments were selected based on data from previous work (Lindstrom et al. 2014) with the exception of changing the initial voltage control setting to be a current control setting. This modification allows the molecular load of interest to be electrically attracted to the lance array in greater quantities because there is current flowing continuously in the solution as opposed to the voltage control setting that sees an exponential decay in current flow in the solution. Those treatment samples that were injected more than once were incubated without changing media for 1 h before the sample was injected again. This refractory period has been shown in previous testing to prevent excessive stress to target cells.

As a convention, specific treatment sample types will be designated by cell type, current control setting used during injection, and the number of times the sample was injected. For instance, Fibro 1.5 mA, ×2 refers to a fibroblast treatment sample that was injected with 1.5 mA (used for Input 1), two times.

### Post testing flow cytometry preparation

Following injections, all samples were incubated for 2 h prior to being treated with 0.5 mL of 5× Trypsin (Gibco) and incubated for 5 min for removal of cells from the glass slide. Following treatment with trypsin, samples were given 1.5 mL of DMEM/FBS to deactivate the trypsin. After transferring individual samples to FACS tubes, the samples were centrifuged for 10 min at 2000 rpm. Final preparation for flow cytometry of the samples include decanting supernatants and re-suspending cell pellets in 0.5 mL of PBS.

### Flow cytometry

Quantification of samples was performed using flow cytometry (Attune Acoustic Focusing Flow Cytometer, Life Technologies). Approximately 20,000 events were captured and characterized for each sample. Data extraction was performed using Attune Cytometric 2.1 software (Applied Biosystems, Life Technologies) by facilitating visualization of events, gating of appropriate cell populations, and developing primary level data usable for JMP (SAS) statistical analysis.

### Statistical analysis

Primary level data generated from post-flow analysis in Attune Cytometric software was evaluated in a two-part process. First, the percentage of living, PI positive cells in each sample were calculated according to the following formula:

2 $$\frac{Number\, of\, Living\, PI \,Positive\, Cells\, in \,Sample}{Number\, of\, Living\, Cells \,in \,Sample}$$

Second, data was then screened in JMP for statistical significance using ANOVA test (F-ratio for HeLa study: 21.0098; F-ratio for Fibroblast study: 49.1873) followed by individual student t-tests (α = 0.05).

## Results and discussion: Serial injection experimentation

**Fig. 7:**
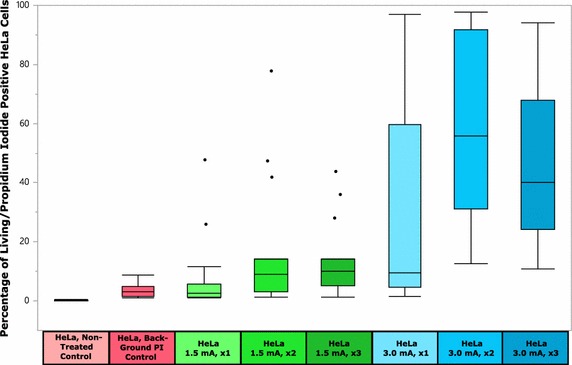
Mean percentage of living/propidium iodide positive HeLa cells for all sample types. Because of the number of statistical relationships that were derived, statistically significant relationships are not noted on the *box plot figure*. For statistical significant relationships, reference Table 3

Figure 7 shows box plot results of the HeLa cell serial injection experiment. Three findings can be seen in these plots and quantitatively represented in Tables 3 and 4. First, the samples receiving 3.0 mA during Input 1 had a higher mean number of modified cells than samples receiving 1.5 mA. The relative maximum for the 3.0 mA samples reached a mean value of 60.47 %, which is nearly four times greater than the relative maximum achieved for the 1.5 mA group. Second, within groups receiving the same current control during Input 1 of the injection process, samples that were injected twice had a higher mean number of modified cells than those samples injected one or three times. Third, within the current control groups, both groups receiving three injections had mean values for PI uptake that were intermediate levels for the current control group.

**Table 3:** P value for HeLa serial injection experiment

Comparison	P value	Comparison	P value
NTC versus BC	0.5813	1.5 mA, ×1 versus 1.5 mA, ×3	0.3578
NTC versus 1.5 mA, ×1	0.2629	1.5 mA, ×1 versus 3.0 mA, ×1	0.0009
NTC versus 1.5 mA, ×2	0.0114	1.5 mA, ×1 versus 3.0 mA, ×2	<0.0001
NTC versus 1.5 mA, ×3	0.0346	1.5 mA, ×1 versus 3.0 mA, ×3	<0.0001
NTC versus 3.0 mA, ×1	<0.0001	1.5 mA, ×2 versus 1.5 mA, ×3	0.6946
NTC versus 3.0 mA, ×2	<0.0001	1.5 mA, ×2 versus 3.0 mA, ×1	0.0381
NTC versus 3.0 mA, ×3	<0.0001	1.5 mA, ×2 versus 3.0 mA, ×2	<0.0001
BC versus 1.5 mA, ×1	0.5299	1.5 mA, ×2 versus 3.0 mA, ×3	<0.0001
BC versus 1.5 mA, ×2	0.0402	1.5 mA, ×3 versus 3.0 mA, ×1	0.0141
BC versus 1.5 mA, ×3	0.1032	1.5 mA, ×3 versus 3.0 mA, ×2	<0.0001
BC versus 3.0 mA, ×1	<0.0001	1.5 mA, ×3 versus 3.0 mA, ×3	<0.0001
BC versus 3.0 mA, ×2	<0.0001	3.0 mA, ×1 versus 3.0 mA, ×2	<0.0001
BC versus 3.0 mA, ×3	<0.0001	3.0 mA, ×1 versus 3.0 mA, ×3	0.0274
1.5 mA, ×1 versus 1.5 mA, ×2	0.1903	3.0 mA, ×2 versus 3.0 mA, ×3	0.0574

**Table 4:** Statistical summary for HeLa cell serial injection experiment

Sample type	Sample size (n)	Mean (%)
HeLa, NTC	24	0.1996
HeLa, BC	24	3.3325
HeLa 1.5 mA, ×1	16	7.3231
HeLa 1.5 mA, ×2	16	16.4594
HeLa 1.5 mA, ×3	16	13.7281
HeLa 3.0 mA, ×1	16	30.9944
HeLa 3.0 mA, ×2	15	60.4720
HeLa 3.0 mA, ×3	15	46.7300

**Fig. 8:**
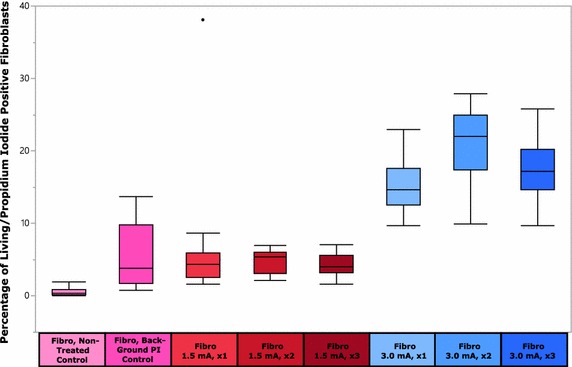
Mean percentage of living/propidium iodide positive fibroblast cells for all sample types. Because of the number of statistical relationships that were derived, statistically significant relationships are not noted on the *box plot figure*. For statistical significant relationships, reference Table 3

Figure 8 shows the box plot results for the serial injection of primary, fibroblasts using the two different current control settings. Two main features are observed in the results. First, the Fibro 3.0 mA, ×2 sample group had the highest mean percent of modified cells for all samples (i.e. 20.97 %), being more than three times greater than the best mean percent in the 1.5 mA group. Second, similar to the behavior seen in the HeLa samples for serial injection, the fibroblasts treated with 3.0 mA also had a relative maximum mean value for the two times injected group.

Tables 5 and 6 provide both statistically significant relationship and summaries for the experiment. Of note, the fibroblasts exhibit lower mean values for the PI delivery than analogous HeLa samples. Also of note, the HeLa samples that were treated with 3.0 mA had a much larger variability in the grouping of data points when compared to the fibroblast 3.0 mA treatment groups.

**Table 5:** P values for fibroblast serial injection experiment

Comparison	P value	Comparison	P value
NTC versus BC	<0.0001	1.5 mA, ×1 versus 1.5 mA, ×3	0.1745
NTC versus 1.5 mA, ×1	<0.0001	1.5 mA, ×1 versus 3.0 mA, ×1	<0.0001
NTC versus 1.5 mA, ×2	0.0028	1.5 mA, ×1 versus 3.0 mA, ×2	<0.0001
NTC versus 1.5 mA, ×3	0.0095	1.5 mA, ×1 versus 3.0 mA, ×3	<0.0001
NTC versus 3.0 mA, ×1	<0.0001	1.5 mA, ×2 versus 1.5 mA, ×3	0.6654
NTC versus 3.0 mA, ×2	<0.0001	1.5 mA, ×2 versus 3.0 mA, ×1	<0.0001
NTC versus 3.0 mA, ×3	<0.0001	1.5 mA, ×2 versus 3.0 mA, ×2	<0.0001
BC versus 1.5 mA, ×1	0.6775	1.5 mA, ×2 versus 3.0 mA, ×3	<0.0001
BC versus 1.5 mA, ×2	0.5598	1.5 mA, ×3 versus 3.0 mA, ×1	<0.0001
BC versus 1.5 mA, ×3	0.2826	1.5 mA, ×3 versus 3.0 mA, ×2	<0.0001
BC versus 3.0 mA, ×1	<0.0001	1.5 mA, ×3 versus 3.0 mA, ×3	<0.0001
BC versus 3.0 mA, ×2	<0.0001	3.0 mA, ×1 versus 3.0 mA, ×2	0.0002
BC versus 3.0 mA, ×3	<0.0001	3.0 mA, ×1 versus 3.0 mA, ×3	0.1438
1.5 mA, ×1 versus 1.5 mA, ×2	0.3646	3.0 mA, ×2 versus 3.0 mA, ×3	0.0286

**Table 6:** Statistical summary for fibroblasts cell serial injection experiment

Sample type	Sample size (n)	Mean (%)
Fibro, NTC	23	0.5383
Fibro, BC	24	5.7713
Fibro 1.5 mA, ×1	16	6.3556
Fibro 1.5 mA, ×2	15	4.9353
Fibro 1.5 mA, ×3	16	4.2588
Fibro 3.0 mA, ×1	16	15.1175
Fibro 3.0 mA, ×2	16	20.9738
Fibro 3.0 mA, ×3	14	17.4550

Key elements shown in the results of both HeLa and fibroblast cells is that the cells respond to the series of injections by having an increase in PI introduction, with the exception of the samples injected 3 times. Two possibilities to explain the decrease in PI observed in three times injected samples are related to physiologic responses to LAN. One possibility is that the 1 h rest period given to the cells following injection is not long enough for cellular structures to recover from the injection event. Even though LAN has been shown to be mild in terms of cell viability after a single injection event (Lindstrom et al. 2014), it is possible that after three injections, that a longer rest period is required in order to reduce cellular stress and increase cell survival rates. A second possibility is related to the effects on the cellular adhesion to the glass slide following injection. Perhaps even though cells are able to maintain adhesion to the glass slide at high rates (as shown in the first portion of this work), that residual strain collects in the CAM structures and after three times of injection, the CAM can no longer adhere to the glass, resulting in a decreased observed number of PI in the sample. Suggested here is further investigation into what effects the rest period duration has on the ability of cultured cells to withstand multiple injection events. Treatment of the substrate for different cell adhesion was not explored since all of the experiments presented here utilized untreated glass coverslips. Future studies could also include treatment of the glass prior to seeding the cells to determine how various adhesive forces due to cell attachment may influence the efficiency of injection with various speeds.

Despite the decreases seen in the samples injected three times, the samples are still exhibiting relatively high levels of PI modification. For instance, the fact that the primary fibroblasts had a lower number of cells modified than the HeLa cells is not unexpected, particularly given the fact that BJ (ATCC^®^ CRL-2522™) fibroblasts are difficult to modify (Avci-Adali et al. 2014). Even so, non-optimized delivery of PI still reached levels of 20.97 %. Furthermore, it is clear that the magnitude of the current control used in Input 1 has a dramatic impact on PI delivery and warrants further exploration.

## Discussion: General

Since PI is able to diffuse from the cytoplasm to the nucleus, these data do not distinguish between cytoplasmic and nuclear injections, but simply identify the ability of the lance array to deliver PI into the cell. However, previous work using a single-lance nanoinjector for delivery of a transgene into fertilized mouse eggs demonstrated that the lance produces an intracellular electroporetic effect allowing cytoplasmic positioning of the lance to still yield nuclear delivery (Wilson et al. 2013).

The combination of results obtained for the speed of injection and the serial injection testing of the LAN represent three major findings. First, cell cultures are able to adhere to the glass slide used for staging the injection process much better when the speed of injection is reduced below 0.16 mm/s. Second, during serial injection testing, samples treated with 3.0 mA during Input 1 and injected twice appear to have the greatest mean percent of living, PI positive cells. Third, the cell type appears to have an effect on how well cells are modified by PI during the injection process, with HeLa cells performing better than primary, neonatal fibroblasts.

All three of these findings are viewed as particularly important milestones in regards to LAN because of how influential they are in establishing higher transfection rates. As noted earlier, there are several biotechnologies that have attempted to address the challenge of molecular delivery by non-viral means but are still plagued with transfection efficiency issues, which varying widely because of intrinsic weaknesses that are part of the technology or because of the cell type being transfected (Mellott et al. 2013).

Traditionally, viruses have been the benchmark for which transfection efficiency is measured. However, they fall short of meeting critical design requirements for robust transfection, particularly in preparation for clinical application. Adenoviruses, Adeno-associated viruses, and lentiviruses are all considered to have high transfection rates in a wide range of cell types (Gardlik et al. 2005; Silman and Fooks 2000). Unfortunately, adenoviruses are immunologically inflammatory which can be life-threatening (Bessis et al. 2004; Ritter et al. 2002), adeno-associated viruses can cause insertional mutagenesis which can be cytotoxic (Deyle and Russell 2009; Monahan and Samulski 2000), and lentiviruses cause immunologic responses and insertional mutagenesis (Follenzi et al. 2007; Hacein-Bey-Abina et al. 2003, 2008; Matrai et al. 2010; VandenDriessche et al. 2002). While retroviruses are useful in CNS (central nervous system) targets (Gardlik et al. 2005; Verma and Somia 1997), the risk of insertional mutagenesis is quite high (Gardlik et al. 2005). Furthermore, viruses in general are limited in their effectiveness because of the limited pay load capacity (≤10 kbp) (Gardlik et al. 2005).

In contrast, LAN is able to by-pass many of these short-comings. First, LAN does not utilize protein vehicles which could cross-react with the immune system, thereby removing immunologic response issues. Second, LAN creates relatively large pores in target cells (1–2 μm diameter), allowing for large molecular loads to enter, thus reducing the concern of not having sufficient pay load capacity. Third, LAN is compatible with gene editing tools such as CRISPR-Cas9 that mitigate concerns regarding insertional mutagenesis. While the same can be said of viruses if re-programmed to remove self-insertional mechanisms, LAN does not have the same preparatory work as viral delivery because insertional mutagenesis in the context of LAN is only an element directly related to the molecular load type, not LAN as a delivery method. To improve the success rate of delivery using LAN, further work could include variations in initial current control settings, variations in pulsed voltage protocols, concentrations of the injection material, and types of molecules delivered.

## Conclusion

Effectively placing molecular loads into target cells without threatening the cell’s survival is the overall goal of transfection biotechnologies. One non-viral method presented in this work is known as LAN, a MEMS based device that relies on physical interaction with target cells and electrical direction of molecular loads. Shown in two sequential experiments is the effect that the speed of injection and the ability to inject cells repeatedly have on target cells. In the speed of injection investigation, it was shown that slower injection speeds improve the number of cells still adherent following injection, reaching a peak mean of 99.3 % at 0.08 mm/s injection speed. Using these results, serial injection testing with HeLa 229 cells and BJ(ATCC^®^ CRL-2522™) cells (neonatal, primary fibroblasts) were conducted by injecting samples multiple times (1, 2, and 3 times) at two different current control settings (1.5 and 3.0 mA). Results show that HeLa cells treated with 3.0 mA and injected twice (×2) had the greatest mean PI uptake of 60.47 % and that neonatal fibroblasts treated with the same protocol reached mean PI uptake rates of 20.97 %. Together these findings help to establish LAN as a method that can obtain modification rates comparable to other transfection technologies.
